# A Notch signal pathway related gene signature predicts overall survival in colorectal cancer

**DOI:** 10.1186/s12876-025-04292-1

**Published:** 2025-10-08

**Authors:** Mingyou Dong, Zhihao Ding, Xinyi Liang, Meng Gao, Mengqi Fan, Xueying Peng, Lusheng Liao, Xiaodong Zhang

**Affiliations:** 1 The Key Laboratory of Molecular Pathology (For Hepatobiliary Diseases) of Guangxi, Affiliated Hospital of Youjiang Medical University for Nationalities, Baise, 533000 Guangxi China; 2 Modern Industrial College of Biomedicine and Great Health, Youjiang Medical University for Nationalities, Baise, China; 3 Hubei Key Laboratory of Cell Homeostasis, College of Life Sciences, Wuhan University, Wuhan, 430072 Hubei China; 4 Department of Reproductive Medicine, Affiliated Hospital of Youjiang Medical University for Nationalities, Baise, 533000 Guangxi China

**Keywords:** Colorectal cancer, Notch pathway, Gene signature, Overall survival, Cell phenotype

## Abstract

**Background:**

Colorectal cancer (CRC) is a prevalent malignancy worldwide, characterized by high morbidity and mortality rates. The notch signaling pathway plays a dual role as both a tumor suppressor and promoter during embryonic development. However, the precise role and underlying mechanisms of notch signal pathway-related genes in CRC remain unclear.

**Methods:**

In this study, a prognostic signature for CRC was established using CRC datasets from The Cancer Genome Atlas (TCGA) as the training datasets and two Gene Expression Omnibus (GEO) datasets for external validation. The limma R package was first used to screen the differentially expressed genes (DEGs) of the Notch pathway in CRC from TCGA datasets. The univariate Cox regression analysis identified 12 Notch pathway related genes (NPRG) that were significantly associated with overall survival (OS) in CRC. Next, the least absolute shrinkage and selection operator (LASSO) and multivariate Cox regression analysis were used to establish a 2-gene prognostic signature (HEYL & WNT5A). Receiver operating characteristic (ROC) curve showed that the 2-gene prognostic signature could effectively predict the 1-, 2-, and 3-year survival time of CRC patients. In addition, the prognostic value of the 2-gene signature was validated in two GEO CORD&READ cohorts. Gene ontology (GO) and Kyoto Encyclopedia of Genes and Genomes (KEGG) analyses were performed to explore the signaling pathways and cellular processes associated with the 2-gene signature. A nomogram for predicting the OS of CRC patients was constructed by integrating clinical characteristics and the 2-gene NPRG signature, followed by validation using calibration curves and decision curve analysis (DCA) plots.

**Results:**

The NPRG prognostic signature for CRC was established. HEYL and WNT5A were identified as hub genes of the Notch pathway in CRC. HEYL was significantly up-regulated in the high-risk group, while WNT5A was significantly up-regulated in the low-risk group. High expression of HEYL was associated with a poorer prognosis of CRC. Furthermore, Knockout of HEYL significantly inhibited the proliferation of CRC cells in HCT116 and RKO cell lines in vitro experiments.

**Conclusions:**

Our research suggests that the 2-gene signature described here can serve as a reliable prognostic biomarker for predicting the prognosis of CRC patients.

**Supplementary Information:**

The online version contains supplementary material available at 10.1186/s12876-025-04292-1.

## Background

According to recent global cancer statistics, there were about 1.9 million new colorectal cancer (CRC) cases and 935,000 deaths in 2020, accounting for about one-tenth of all cancer cases and deaths. Overall, colorectal cancer was the third most common cancer type, and the second most common cause of cancer-related death [1]. The evolution of colorectal cancer involves multiple stages, with the TNM staging (guided by the tumor-lymph node metastasis (TNM) system) still being the most classic [2]. It is worth noting that the development of colorectal cancer is complex and is associated with many genes. Evidence suggests that several factors, including genetic and environmental factors, intestinal colonies, dietary habits, and nutrition, play a significant role in CRC development [3, 4]. However, CRC diagnosis is hampered by the lack of early symptoms.

Over the years, there have been significant advances in surgical treatment of CRC, and the last 15 years have witnessed a dramatic change in CRC treatment largely due to the invention of several new therapeutic agents [5]. For example, immunotherapy has now become a highly effective cancer treatment method [6]. Nevertheless, although the treatment and prognosis of colorectal cancer have improved significantly as a result of combination therapy, the prognosis is still poor, especially in metastatic states [7]. Moreover, CRC is a highly heterogeneous disease, and studies have shown that the location of tumor growth is correlated with age and gender [8]. However, despite the numerous studies on colorectal cancer, its migration mechanism has not yet been fully elucidated [9].

It is well known that there are some highly conserved pathways in evolution. Comparative analysis of the genomes of bilaterals and early branches of metazoa has revealed four major signaling pathways: Wnt, Notch, transforming growth factor-beta (TGF-beta), and Hedgehog pathways [10]. This study explored the Notch signaling pathway, with the overarching goal of identifying biomarkers for colorectal cancer.

The Notch gene was first observed in the mutation of Drosophila morgana in 1917, and was first isolated and characterized in 1983 [11]. The Notch signaling pathway is highly conserved in evolution and has been shown to regulate many cellular processes, including cell proliferation, stem cell maintenance, differentiation, and death [12]. Notably, Notch signaling is an intercellular communication system in which the Notch ligand on the membrane of one cell (signal-presenting cell) interacts with the transmembrane Notch receptor on the membrane of the adjacent cell (signal-receiving cell). NICD is then cut and migrates to the nucleus where it binds to DNA binding protein CSL (CBF1/Suppressor of Hairless/LAG1; Also known as RBPJ) and Mastermind-like proteins (MAML), which act as transcription factors regulating downstream pathways [13]. The non-canonical Notch signal can be initiated in several ways: with or without a non-standard ligand, without the Notch signal, and even without CSL in some cases [14]. Although the function of Notch receptors is simple, Notch can exert different effects, such as promoting or inhibiting cancer [15]. For example, Notch is carcinogenic in T-cell malignancies (T-all and T-cell Hodgkin’s lymphoma), but has a tumor suppressive effect in acute myeloid leukemia, B-cell acute lymphoblastic leukemia, and chronic monocytic leukemia [16]. In addition, loss-of-function mutations in NOTCH1 were found in a subset of head and neck squamous cell carcinoma (HNSCC), thus, NOTCH1 is considered as a tumor suppressor in the oropharyngeal epithelium. However, NOTCH1 gain-of-function mutations were also found in HNSCC, which suggests that Notch acts as an oncogene in HNSCC [17]. Notch can be both an oncogene and a tumor suppressor gene in different cells of the same tumor in small-cell lung cancer (SCLC) [18]. Currently, clinical therapies targeting Notch are still being developed, and fusion antibodies to DLL4-FC have been used to treat SCLC models [19]. Moreover, some DLL4 antibodies, such as MEDI0639, are undergoing phase I clinical trials [20]. However, the role of Notch in colorectal cancer has rarely been explored and the Notch signaling pathway has not been considered in the construction of CRC prognostic features.

This study screened colorectal cancer genes associated with the Notch pathway using data retrieved from public databases. Next, a prognostic multigene signature of Notch related differentially expressed genes (DEGs) was established using Lasso-Cox analysis. It should be noted that the identified tumor markers can not only monitor the prognosis, but also predict the clinical status of colorectal cancer patients, including tumor occurrence and progression. The constructed model was then validated using multiple datasets from The Cancer Genome Atlas (TCGA) and Gene Expression Omnibus (GEO) databases. Finally, knockout cell lines were established using CRISPR/Cas9 system to explore whether the HEYL marker was consistent with the prediction in vivo.

## Methods

### Dataset collection

The raw RNA-sequencing (RNA-seq) datasets and the clinical characteristics of CORD&READ cohorts were downloaded from the TCGA database. GSE33133 and GSE38832 datasets were downloaded from the GEO database for external validation. In addition, the 32 Notch pathway related gene sets (HALLMARK_NOTCH_SIGNALING) were extracted from the Molecular Signatures database v7.1 (MSigDB) [21]. The details of the 32 genes were presented in Table S1.

### Identification of DEGs

The dplyr R package was used to normalize all CRC gene expression data downloaded from TCGA and GEO databases. The genes without expression data were excluded from the analysis. Notably, 647 CORD&READ samples and 51 normal samples were retrieved from TCGA. First, the differentially expressed genes (DEGs) were identified using the limma R package [22], followed by screening out DEGs associated with the Notch pathway (FDR < 0.05). Next, Gene Ontology (GO) and the Kyoto Encyclopedia of Genes and Genomes (KEGG) pathway enrichment analyses were performed in clusterProfiler R package [23] to evaluate the function of these Notch pathway-related DEGs (FDR < 0.05 and |log2FC| >1).

### Construction of a prognostic Notch pathway-related gene (NPRG) signature

Univariate Cox analysis was performed to screen for prognosis-related NPRG (OS, *p* < 0.05). Then, the least absolute shrinkage and selection operator (LASSO) regression analyses was performed in glmnet R package to select the key genes from prognosis-related NPRG. The value of penalty parameter (λ) corresponding to the lowest partial likelihood deviance was used to select the best model by 10-fold cross-validation. We got a list of genes with non-zero beta coefficients. Finally, a stepwise multivariate Cox regression analysis was used to construct a prognostic risk model. The formula for the signature-based risk score was as follows: Score = (mRNA1 regression coefficient* expression level of mRNA1) + (mRNA2 regression coefficient * expression level of mRNA2) + … + (mRNAn regression coefficient * expression level of mRNAn). The Kaplan-Meier method was used to analyze the overall survival between low-risk and high-risk groups. Time-dependent receiver operating characteristic (ROC) curves were generated in survival ROC R package to evaluate the prediction efficiency of the established risk score model. Moreover, scatterplots were used to show the distribution of risk scores, and the correlation between risk scores and OS. Principal component analyses (PCA) in stats R package’s prcomp function and t-distributed stochastic neighbor embedding (t-SNE) in Rtsne R package were utilized to verify the clustering conditions of the established gene signature. Finally, two external GEO datasets (GSE33133 and GSE38832) were used to verify the effectiveness of the model.

### Univariate and multivariate Cox regression analyses, and construction of a nomogram

Univariate and multivariate prognostic Cox regression analyses were performed to evaluate whether the risk score is an independent prognostic factor. A prognostic nomogram for predicting the 1-, 3-, and 5-year OS of colorectal cancer patients was established using rms R package based on the age, gender, stage, T stage, N stage, and risk score. The concordance index was then used to assess the accuracy of the nomogram, whereas the calibration plot was used to determine the prediction efficiency of the nomogram. Time-dependent ROC curves were generated to show the 1-, 3-, and 5-year overall survival. Finally, a concordance index plot and a standard net benefit plot were used to evaluate the reliability of the nomogram.

## Enrichment analyses

DEGs between high risk and low risk patients in the cohort were identified using the limma R package. GO and KEGG analyses were performed using the ClusterProfiler R package [23] to evaluate the biological functions of DEGs in the two groups [FDR ≤ 0.05, log2FC > 1].

### Plasmid, cell culture, and plasmid transfection

The plasmids used in this study were lentiCRISPRv2 (Addgene, #52961), pMG2G (Addgene, #12259), and psPAX2 (Addgene, #12260), whereas the cells used were human CRC cell lines (HCT116 and RKO cells) and the human embryonic kidney 293 T cell line (HEK293T). HEK293T cells were cultured in DMEM (HyClone, Logan, Utah, USA) supplemented with 10% FBS (Gibco, Carlsbad, CA, USA) and 1% penicillin-streptomycin (HyClone, Logan, Utah, USA) at 37°C in 5% CO_2_. On the other hand, HCT116 and RKO cells were cultured in McCoy’s 5 A medium (HyClone, Logan, Utah, USA) supplemented with 10% FBS and 1% penicillin-streptomycin at 37°C in 5% CO_2_. Notably, all plasmids were transfected with poly-ethylenimine (PEI; Polysciences) according to the manufacturer’s instructions.

### CRISPR/Cas9 knockout (KO) cell lines

*HEYL* knockout cell lines were established using the CRISPR/Cas9 system as previously described [24]. Single-guide RNA (sgRNA) was designed online (*Guide design resources - Zhang Lab (zlab.bio)*) and transformed into lentiCRISPRv2. The sgRNA sequence of *HEYL* was 5’-ccaagcgtcgcaattcagaa-3’. Briefly, the lentiCRISPRv2 linked sgRNA was co-transfected with pMD2.G and psPAX2 in HEK293T cells. *HEYL* knockout cell lines were then selected using puromycin (1 µg/mL) 72 h after infection. Finally, HEYL−/− HCT116 and HEYL−/− RKO cell lines were constructed.

### Cell viability and colony formation assay

Cell Counting Kit-8 (CCK-8) assays were performed to explore the cellular viability WT and KO cells. Briefly, WT and KO cells were seeded into 96-well plates (10^3^ cells/well). After 24 h, the medium of each well was replaced with 90 µL fresh medium mixed with 10% CCK-8 reagent. Next, cells were cultured at 37℃ for 1 h, followed by measuring the absorbance at 450 nm. The colony formation assay was performed as previously described [25]. Cells (500 cells/well) were first seeded in a six-well plate. After 12 days, clones were stained with crystal violet and photographed after washing.

### Statistical analyses

All statistical analyses were performed using R software (version 4.0.5). The chi-square test was used to analyze the risk scores and clinical characteristics, whereas the Kaplan–Meier test and the log-rank test were used to evaluate OS in high-risk and low-risk patients. Univariate and multivariate Cox regression analyses were performed to explore the association between risk score and OS. *p* < 0.05 was considered statistically significant and all P values were based on two-sided statistical tests.

## Results

### Screening of DEGs

**Fig. 1:**
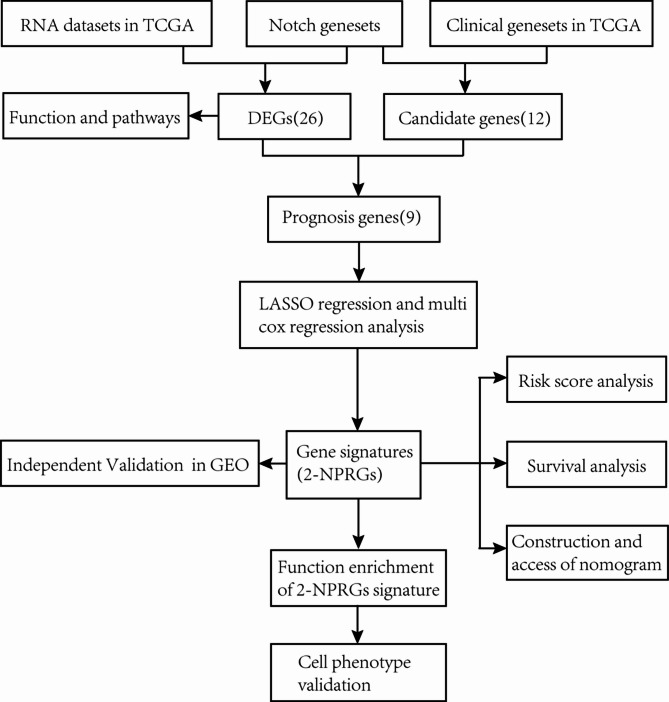
The flow of the study

**Fig. 2:**
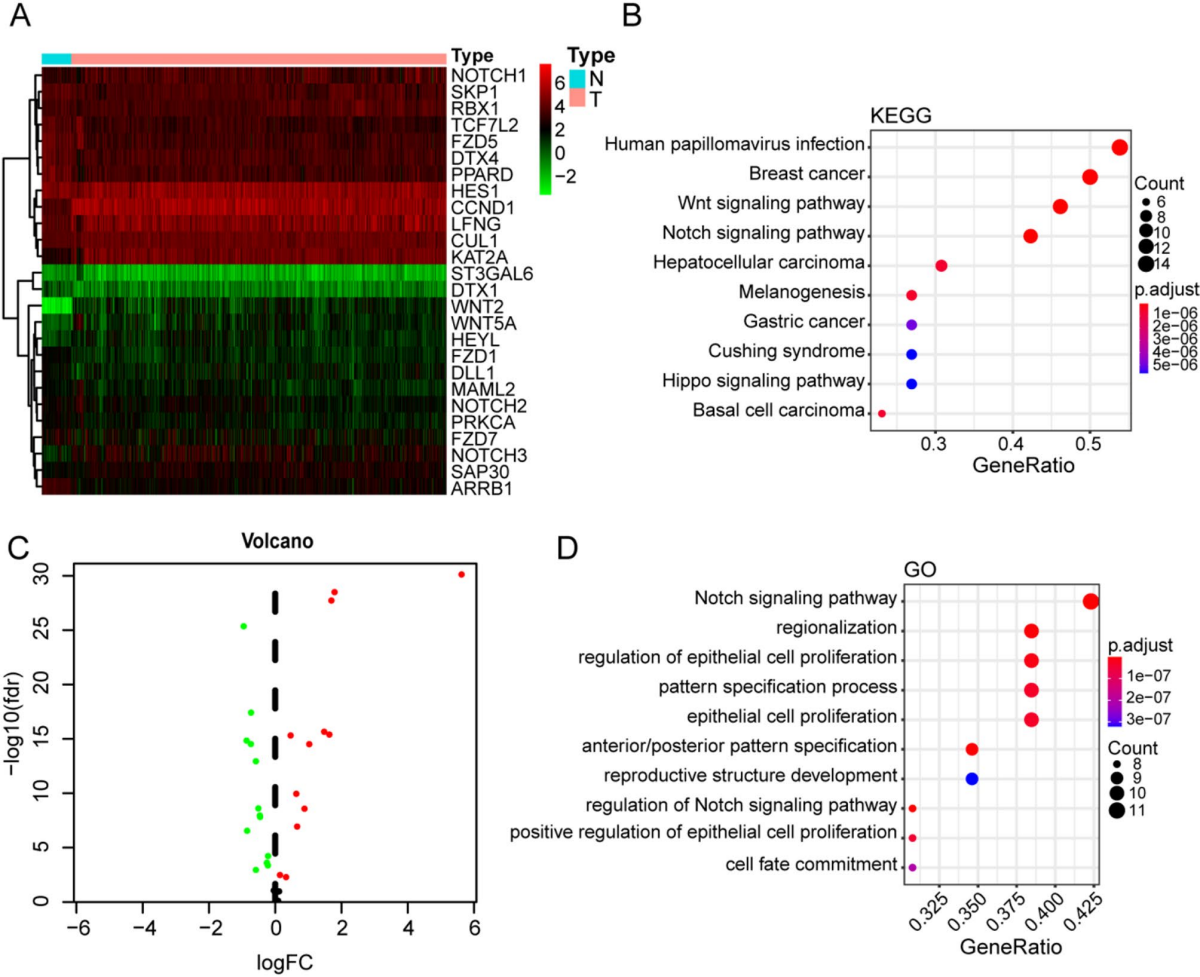
Identification of Notch pathway-related DEGs. **A** Expression heatmap of DEGs. **B** Volcano map of DEGs. **C** GO enrichment analyses of DEGs. **D** KEGG enrichment analyses of DEGs

Figure 1 shows the flowchart of this study. A total of 647 patients from CORD&READ TCGA cohorts were included in the study. Results showed that 26 out of the 32 Notch pathway-related genes between the 51 normal and 647 colorectal cancer samples met the screening standard (*p* < 0.05), including 12 upregulated and 14 downregulated genes (Fig. 2A, B). GO enrichment and KEGG enrichment analyses were then performed to evaluate the function of these DEGs. GO enrichment analysis (Fig. 2C) showed that the DEGs were mainly involved in HPV infection, Wnt, and Notch pathways. On the other hand, KEGG enrichment (Fig. 2D) showed that the DEGs were mainly enriched in the Notch pathway, regionalization, and the epithelial cell proliferation pathway.

### Construction of NPRG prognostic signature

**Fig. 3:**
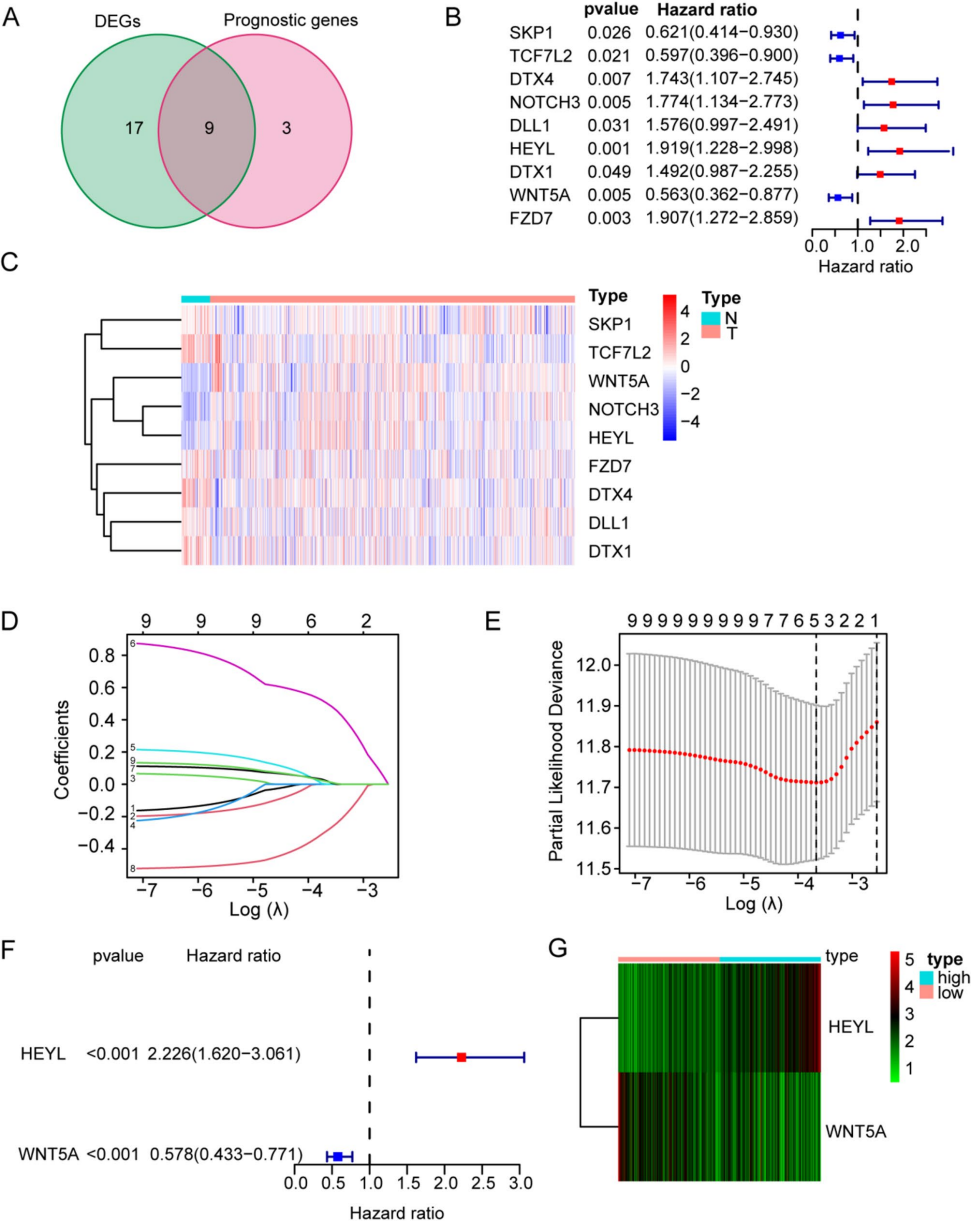
Identification of hub Notch pathway-related gene in the TCGA cohort. **A** Venn plot was used for selection of Notch pathway-related DEGs with prognostic value. **B** Forest plot showing the Prognostic value of 9 NPRGs. **C** Expression heatmap of 9 NPRGs. (**D**-**E**) Least absolute shrinkage and selection operator (LASSO) regression analyses was used for selection of hub NPRGs. **F** Multivariate cox analyses were used for selection of hub NPRGs. **G** Expression heatmap of hub NPRGs

Among the 32 Notch pathway-related genes (NPRG), univariate Cox proportional analyses identified 12 candidate prognostic genes that were significantly associated with OS (*p* < 0.05). The Venn plot diagram showed that nine of the 12 genes exhibited significantly different expression levels between normal and tumor tissues (Fig. 3A). Notably, six genes (*DTX4*,* NOTCH3*,* DLL1*,* HEYL*,* DTX1*, and *FZD7*) were considered as the risk genes, whereas three genes (*SKP1*,* TCF7L2*, and *WNT5A*) were considered as the protective genes according to the value of hazard ratio (HR) (Fig. 3B). Nine candidate NPRGs were significantly differentially expressed between normal and colorectal cancer tissues (Fig. 3C). To avoid over-fitting, five NPRGs were retained by LASSO regression analysis with the lowest partial likelihood deviance (Fig. 3D, E). Then, From the multivariate Cox analyses results, only two genes were selected to establish a gene signature (*p* < 0.001) (Fig. 3F). Next, the risk scores of the signature were used to predict prognosis in colorectal cancer. The risk scores of the signature were calculated according to the following formula:

Score = (−0.54811* expression level of WNT5A) + (0.800397* expression level of HEYL). Patients were then stratified into a high-risk group (*n* = 216) or a low-risk group (*n* = 217) according to the median cut-off value of risk scores. HEYL expression was significantly up-regulated in the high-risk group, while WNT5A expression was significantly up-regulated in the low-risk group (Fig. 3G).

### Evaluation of NPRG signature in TCGA dataset

**Fig. 4:**
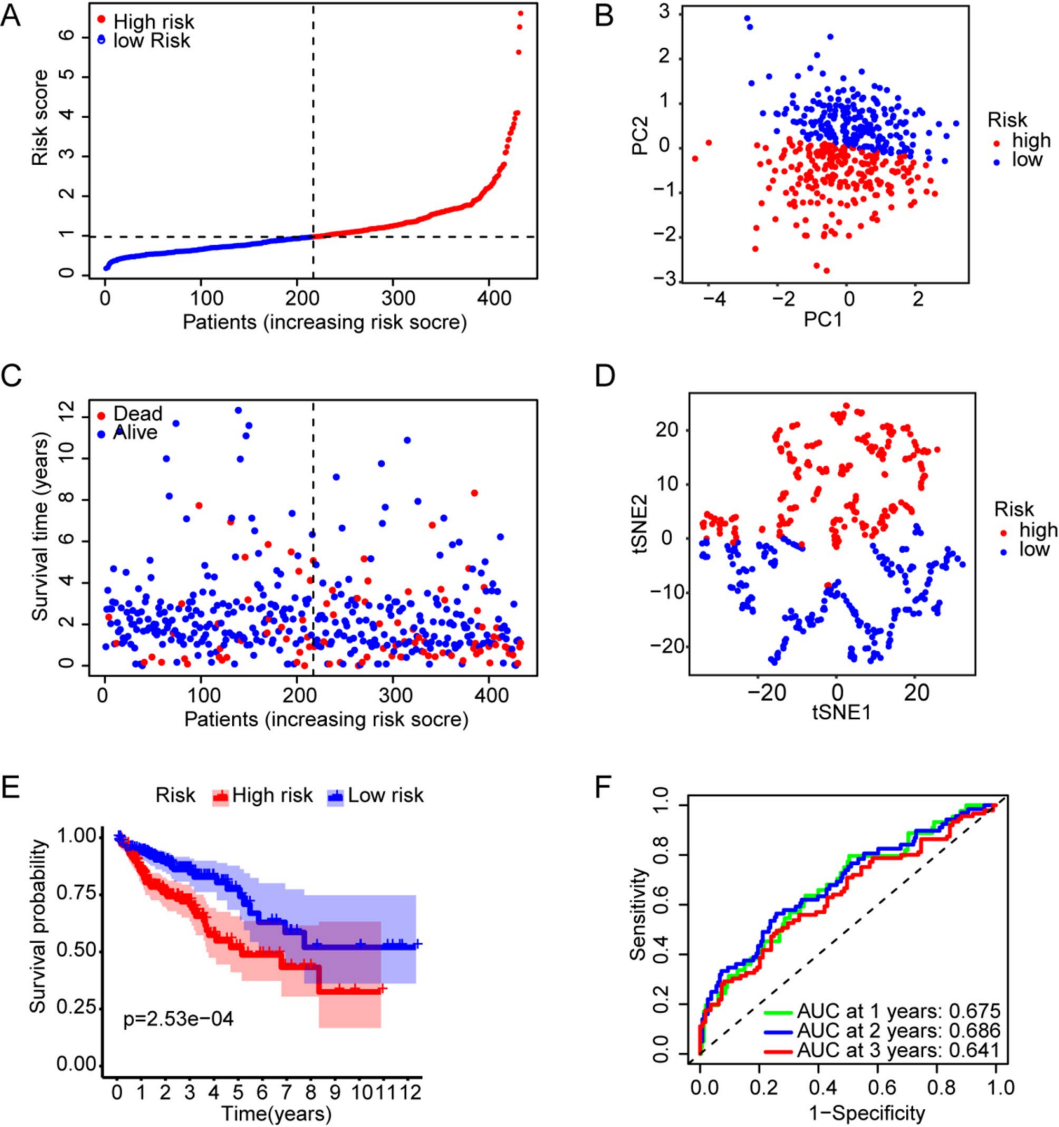
Construction and evaluation of a two gene signature in the TCGA cohorts. **A** Patients were classified by risk scores as high-risk or low-risk. **B** PCA analyses for the TCGA dataset. **C** The distribution of OS status and OS between high-risk and low-risk patients. **D** t-SNE analyses for the TCGA dataset. (E) Kaplan–Meier curves for OS in the two risk groups. (F) Time-dependent ROC curves validation for the risk scores

In the TCGA dataset, CRC patients were divided into high-risk and low-risk groups based on the median value of the risk score (Fig. 4A). There were more deaths in the high-risk group of patients compared to the low-risk group of patients (Fig. 4C). The results of PCA and t-SNE analysis showed that the high-risk and low-risk groups were significantly divided into two different regions (Fig. 4B and D). Kaplan-Meier survival curves showed that patients in the high-risk group had shorter OS compared to patients in the low-risk group (***p*** = 2.53e-04; Fig. 4E). The predictive performance of the risk score for OS was evaluated using time-dependent ROC curves, with results showing that the area under the curve (AUC) was 0.675 at one year, 0.686 at two years, and 0.641 at three years (Fig. 4F).

### Validation of the NPRG signature in independent cohorts

**Fig. 5:**
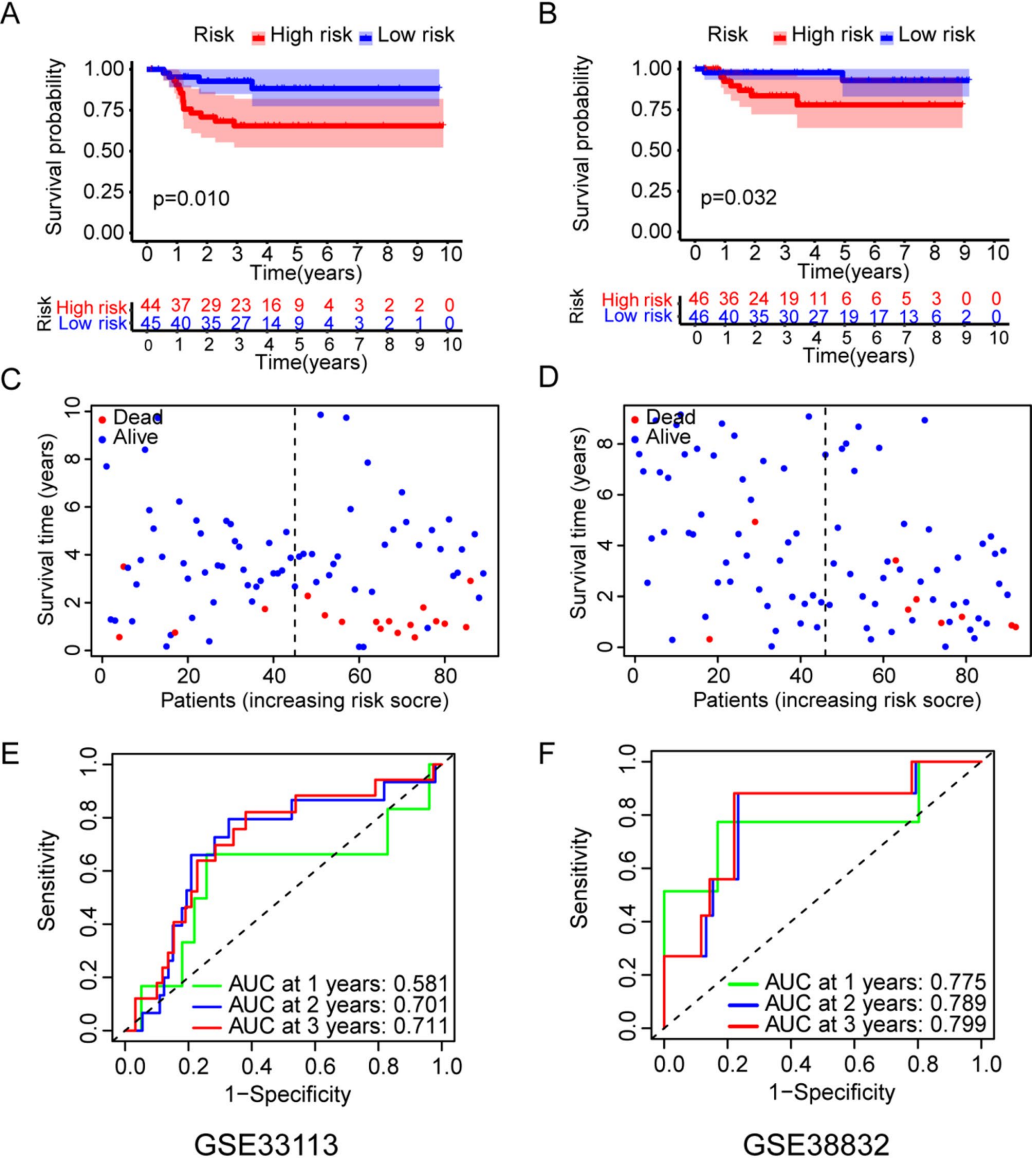
Validation of the NPRG signature in the GSE33133 and GSE38832 datasets. (**A**-**B**) Kaplan-Meier survival between high-risk and low-risk patients in GSE33133 and GSE38832. (**C**-**D**) Kaplan–Meier curves for OS in GSE33133 and GSE38832. (**E**-**F**) Time dependent ROC curves for the risk scores in GSE33133 and GSE38832

In order to analyze the predictive value of NPRG signature in different CRC datasets, the NPRG signature was then verified using two independent external datasets (GSE33113 and GSE38832). The high-risk patients were also more likely to have a shorter OS time compared to low-risk patients in GSE33113 and GSE38832 cohort (Fig. 5A, B). There were more deaths in the high-risk group of patients compared to the low-risk group of patients in two cohort (Fig. 5C, D). The AUC value for predicting OS of CRC patients were 0.581 at 1 year, 0.701 at 2 years, and 0.711 at 3 years in GSE33113 cohort (Fig. 5E). Similar to the GSE33113 dataset, the AUC value for predicting OS of CRC patients were 0.775 at 1 year, 0.789 at 2 years, and 0.799 at 3 years in GSE38832 cohort (Fig. 5F). These results suggest that the NPRG signature can effectively predict the prognosis of colorectal cancer patients.

### The prediction value of NPRG signature in different clinical subgroup

**Fig. 6:**
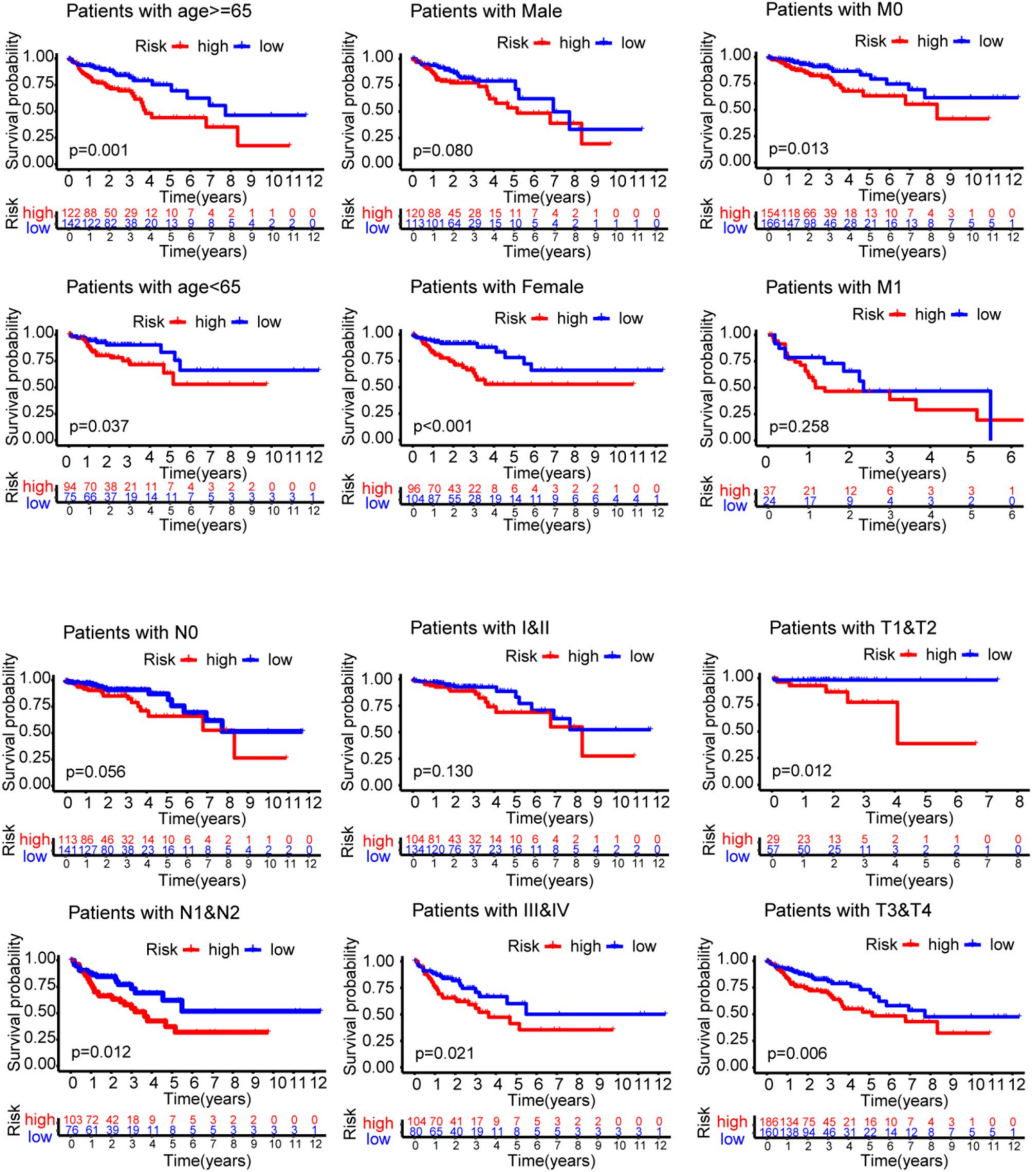
Kaplan-Meier survival plots of NPRG signature in TCGA cohorts with different clinical characteristics

To explore the efficiency of the model for predicting the OS in different clinical subgroup, we divided CRC patients into two subgroups according to different clinical characteristics: age (≤ 65 and > 65), gender (male and female), M stage, N stage, and TNM stage, and analyzed the predictive value of the NPRG signature in different clinical subgroups. The results indicated that, in most subgroups, individuals with high-risk had a worse OS compared to the low-risk individuals (Fig. 6).

### Univariate and multivariate Cox analyses, and nomogram construction

**Fig. 7:**
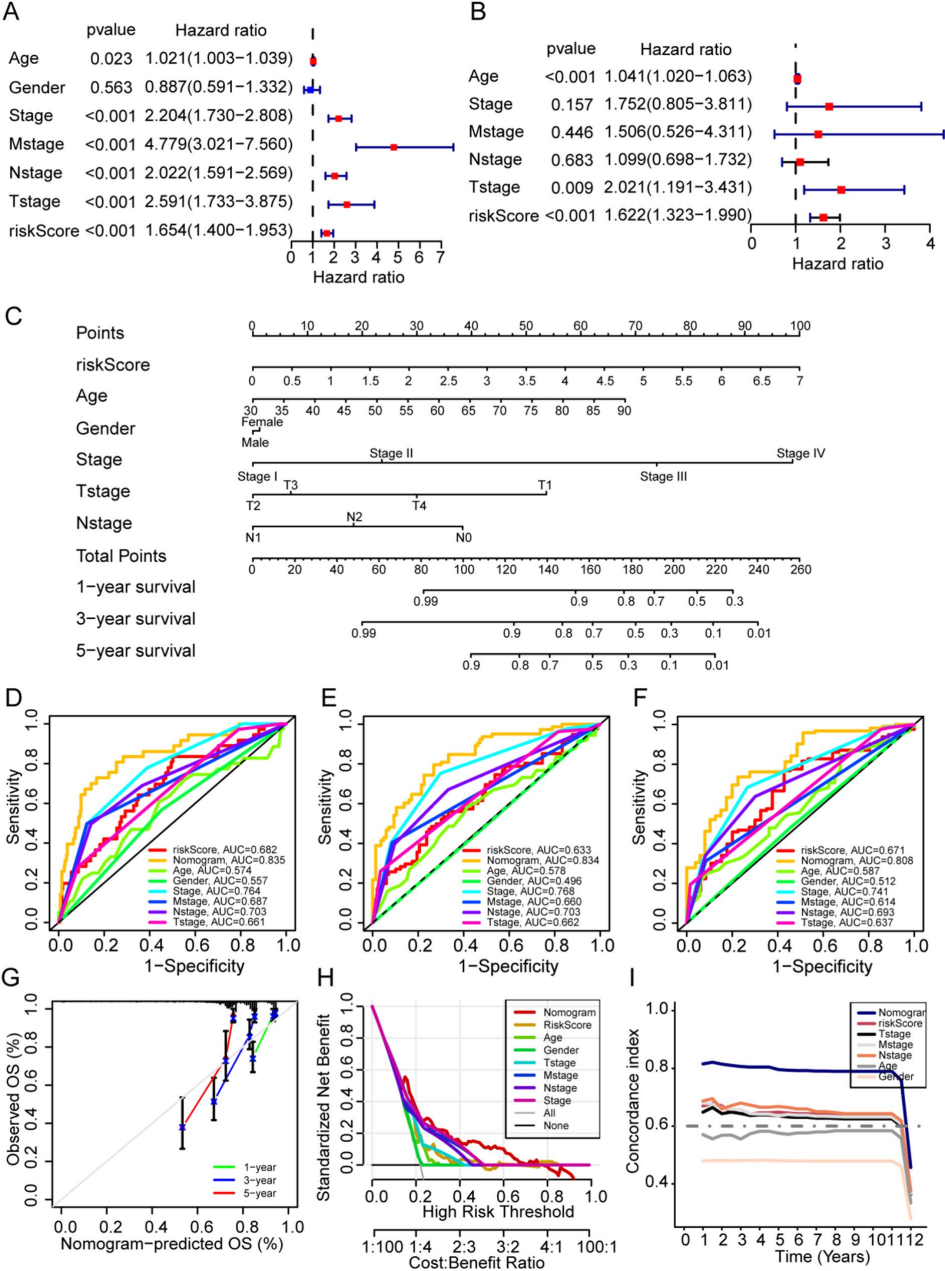
Construction of a nomogram for predicting OS in TCGA cohort. **A** Univariate Cox regression analyses regarding OS in the TCGA cohorts. **B** Multivariate Cox regression analyses regarding OS in the TCGA cohorts. **C** The Nomagram for the OS at 1-, 3- and 5-years. (**D**-**F**) AUC of time-dependent ROC curves for the nomogram of 1-, 3-, and 5-years. **G** Calibration curves for the nomogram in 1-, 3-, and 5-years. (H) DCA plot. **I** Concordance index plot demonstrating the concordance measure of the predictor

Univariate Cox analyses showed that six indicators (Age, Stage, Mstage, Nstage, Nstage, Tstage, and risk score) were significantly associated with the prognosis of CRC patient (Fig. 7A). In addition, multivariate Cox analyses indicated that the three indicators (Age, Tstage, and risk score) were significant independent factors (Fig. 7B). The clinical characteristics of age, gender, stage, Tstage, Nstage and risk score were used to construct a nomogram for predicting the overall survival of CRC individuals at 1-, 3-, and 5-years (Fig. 7C). The time-dependent ROC curves used to predict 1-, 3-, and 5-years OS in CRC patients show that, the nomogram has higher ROC values relative to risk scores and different clinical characteristics. The calibration curve results show that nomogram has a high predictive value (Fig. 7G). A decision curve analysis (DCA) and a concordance index plot were then constructed to evaluate the prediction of the nomogram model (Fig. 7H, I).

### Functional enrichment analyses of the NPRG signature

**Fig. 8:**
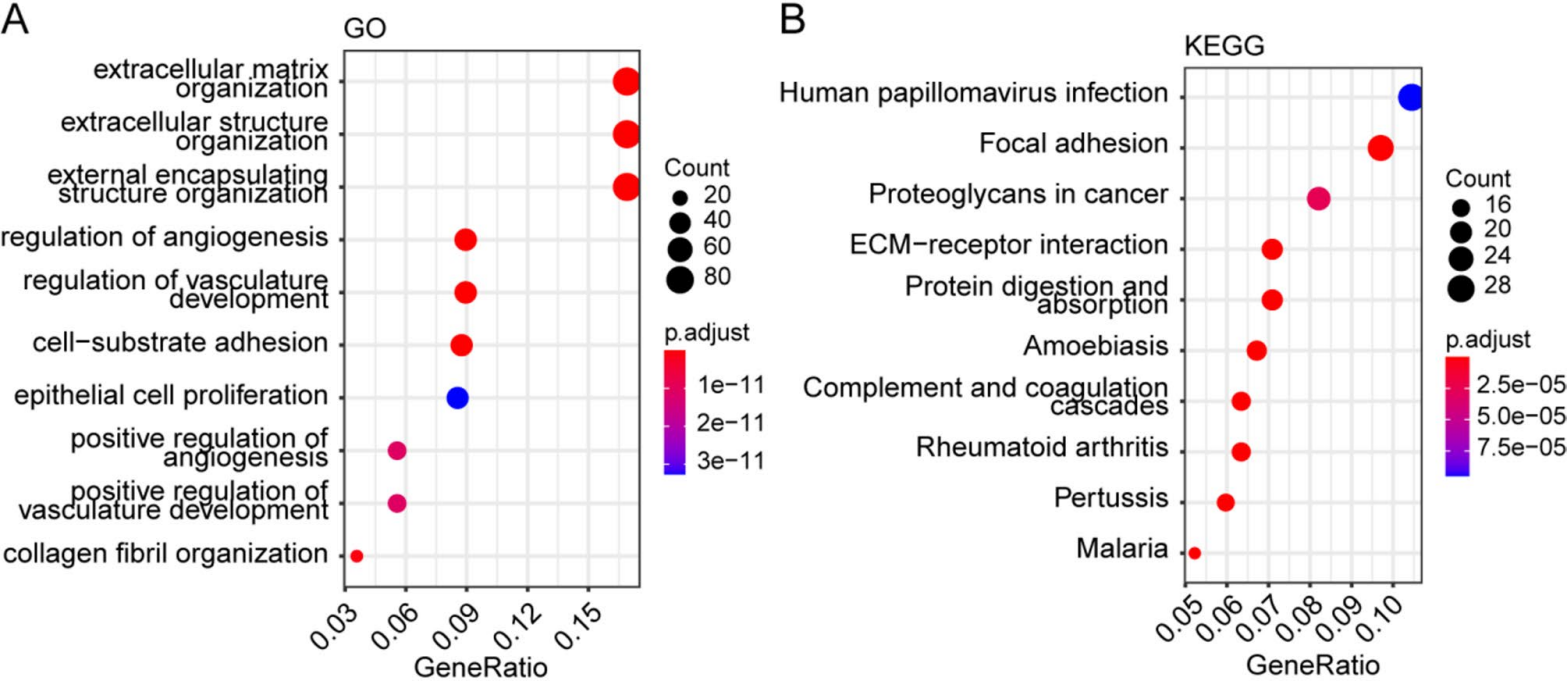
Functional enrichment analysis of DEGs between high and low risk groups. **A** GO enrichment analysis of DEGs. **B** KEGG enrichment analysis of DEGs

Furthermore, the study explored the biological functions of DEGs in the high-risk and low-risk groups. GO enrichment analysis showed that DEGs in each group were mainly enriched in extracellular matrix organization, extracellular structure organization, and external encapsulating structure organization (Fig. 8A). In addition, KEGG pathway analysis revealed that the DEGs were mainly involve in several pathways, including human papillomavirus infection, focal adhesion, proteoglycans in cancer, and extracellular matrix receptor interaction (Fig. 8B).

### Cell phenotype of the HEYL (core gene) knockout cell lines

**Fig. 9:**
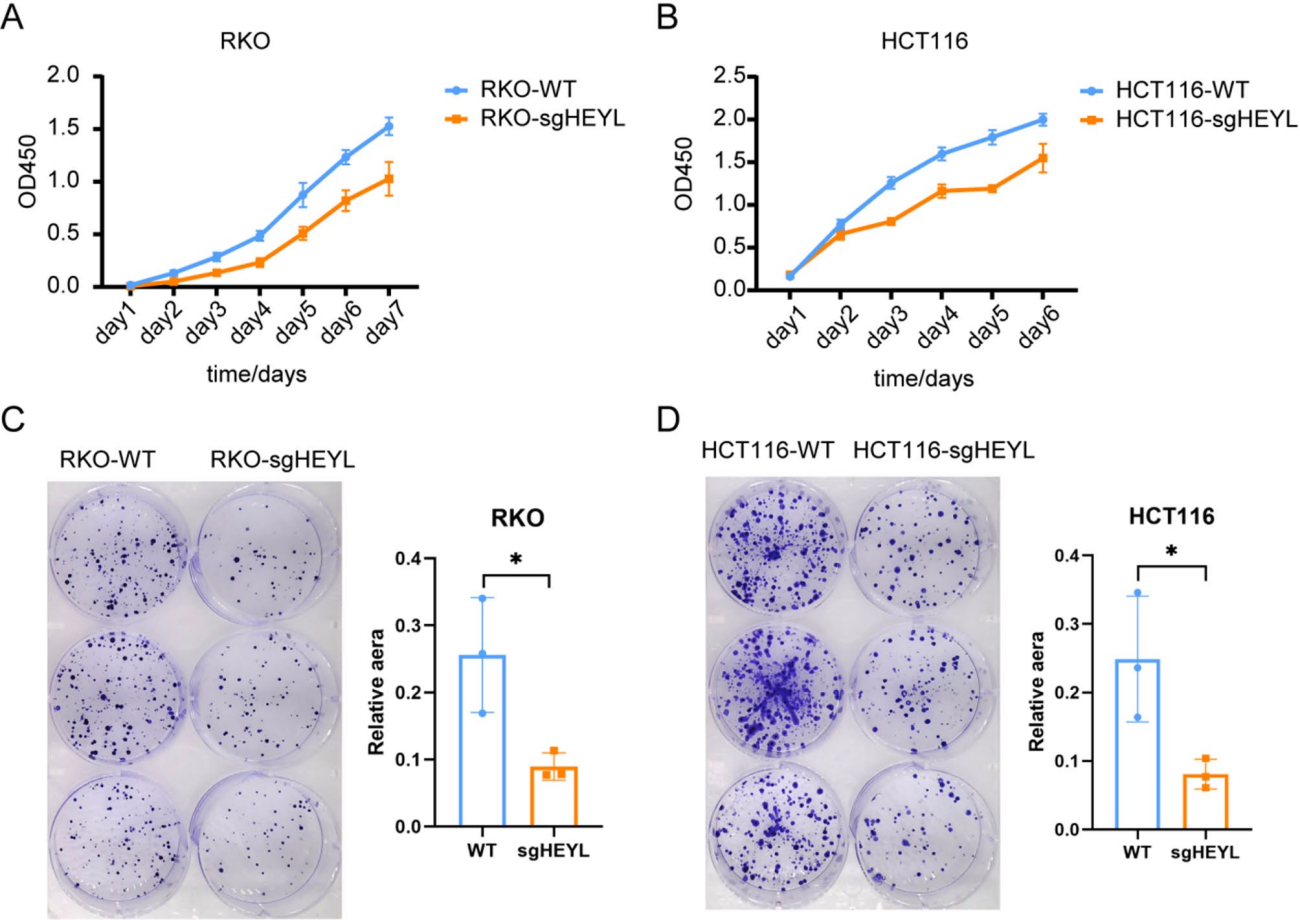
Cell phenotype assay of HEYL knockout cell line. (**A**-**B**) The cellular proliferation of WT and HEYL-KO RKO/HCT116 cell lines (test by CCK8). (**C**-**D**) The colony formation assay of WT and HEYL-KO RKO/HCT116 cell lines (the areas of spots proportion are shown by bar graphs)

It is worth mentioning that a previous study found that *HEYL* is overexpressed in colon cancer. We further validated *HEYL* on other GEO datasets and found significant upregulation across multiple datasets, indicating a poor survival outcome in colorectal cancer patients with high expression of *HEYL*, Furthermore, analysis of the GEO datasets revealed that *HEYL* possessed substantial diagnostic value in colorectal cancer (Fig. S1). Therefore, we knocked out *HEYL* to explore whether it played a similar function in colorectal cancer. CRISPR/Cas9 system was employed to knockout *HEYL* in colorectal cell lines, followed by conventional PCR analysis to determine the effect on CRC cells. Notably, this experiment was performed twice and two KO cell lines (RKO and HCT116) were acquired. Next, cellular proliferation and colony formation assays were conducted to explore the proliferative potential of the two cell lines. The obtained results are shown in Fig. 9. It was evident that the deficiency of HEYL in both HCT116 and RKO cell lines caused low cell viability (Fig. 9A, B) and slow proliferation (Fig. 9C, D), suggesting that *HEYL* plays a positive role in colorectal cancer cells proliferation.

## Discussion

In 2020, colorectal cancer was the third most common cancers and the second leading cause of cancer-related mortality [1]. Cetuximab, targeting epidermal growth factor receptor (EGFR), has been widely used in patients with metastatic colorectal cancer [26]. Moreover, the emergence of molecular targeted therapy has improved the outcome of patients with advanced colorectal cancer. However, although this targeted therapy has achieved remarkable success and specificity, there are significant adverse reactions and individual differences in treatment [27]. Furthermore, given that CRC may have more complex mechanisms, further studies should be conducted to fully elucidate the underlying characteristics of CRC.

The Notch pathway is a highly conserved pathway, which has been extensively studied in a variety of cancers [28]. In colorectal cancer, high NOTCH-1 levels are associated with progression, tumor grade, and metastasis [29]. The Notch signal was 10–30 times higher in cancer stem cells (CSCs) than in common colon cancer cells [30]. Studies have also revealed that Notch prevents apoptosis of CSCs by inhibiting P27 and ATOH1 [31]. In addition, Notch is actively involved in the process of epithelial-mesenchymal transformation (EMT), where epithelial cells acquire mesenchymal phenotypes and migrate to other tissues [32]. Wnt, Notch, BMP, and Hedgehog pathways are involved in normal epithelial differentiation, and changes in these pathways, with the establishment of tumorigenesis, and these pathways have a crosstalk [33]. Notch and WNT signaling pathways are important for maintenance and proliferation of stem cells [34]. Moreover, Notch can promote and inhibit cancer in different tumors or even in the same tumor [18].

Herein, bioinformatics analysis was performed to establish a universal molecular biomarker of CRC, with the conserved Notch pathway being the main focus. The study aimed at identifying some downstream effector molecules that can be used to predict colorectal cancer [35]. Briefly, 32 Notch pathway related genes were collected from a public database, followed by analysis of their expression in normal tissues and colorectal cancer tissues. As expected, 26 Notch pathway related genes were screened out (FDR < 0.01). Results confirmed that nine Notch pathway related DEGs were correlated with the OS of colorectal cancer patients. Moreover, LASSO Cox analysis and a multivariate Cox regression analysis identified a 2-gene signature: *WNT5A* and *HEYL*. Notably, *HEYL* was considered as the risk gene, whereas *WNT5A* was the protective gene.

WNT5A is a crossover molecule and belongs to the Wnt signaling pathway. Previous studies have revealed that the Wnt signaling pathway regulates a variety of processes critical for development, including cell proliferation, differentiation, adhesion, polarity, and motility [5, 36]. Wnt5A is an evolutionally conserved nonclassical Wnt ligand that modulates convergence-elongation (CE), planar cell polarity (PCP), and epithelial mesenchymal interactions during embryonic morphogenesis [37]. The expression of *WNT5A* increases with the progression of cutaneous melanoma, and its expression is considered to be a risk factor for prognosis [38]. One study found that the expression of Wnt5A was also upregulated in cervical cancer tissues compared to adjacent normal cervix tissues, and was positively correlated with lymph node metastasis and recurrence [39]. *WNT5A* is also considered to be a tumor suppressor gene because it inhibits typical Wnt signaling in breast cancer [40]. In nasopharyngeal carcinoma (NPC), overexpression of *WNT5A* in S26 cells significantly induced migration and invasion, whereas stable expression of *WNT5A* in S18 cells significantly reduced migration and invasion [41]. However, several studies have confirmed that the downregulation of *WNT5A* is associated with poor prognosis in CRC and other cancers, suggesting that it has a tumor suppressive effect in CRC [42].

HEYL is an effector downstream of Notch whose expression changes in many diseases. The expression of *HEYL* in human bronchial epithelial cells (HBECs) is reduced compared to normal cells, which can be attributed to the impaired differentiation ability in chronic obstructive pulmonary disease (COPD) [43]. In hepatocellular carcinoma, epigenetic silencing of *HEYL* expression caused by DNA hypermethylation is directly associated with the loss of *HEYL* expression [44]. Notably, *HEYL* is excluded from the nucleus of prostate cancer cells, but not from adjacent tissues [45]. Analysis of myogenic protein promoter activity showed that HEYL and Hes1 synergistically inhibited myogenic differentiation [46]. A previous study reported that overexpression of *HEY1* is a poor prognostic factor in all CRC patients [47]. Therefore, although *HEYL* plays a role as a tumor suppressor gene in liver cancer, chronic obstructive pulmonary disease (COPD), and prostate cancer, it is overexpressed in CRC, suggesting a tumor promotion role.

In this study, all patients in the training set were divided into two groups according to the median risk score and then the 2-gene signature was used to evaluate the OS. The PCA and the t-SNE plot showed a perfected distinction, and the OS of the low-risk group was better than that of the high-risk group. The ROC analysis indicated the efficiency of the 2-gene signature in predicting the 1-year, 2-year, and 3-year OS of CRC. Finally, the efficiency of the 2-gene signatures was validated using two external cohorts, GSE33133 and GSE38832.

The multivariate Cox regression analyses demonstrated that the 2-gene NPRG signature risk score was an independent predictor of OS in colorectal cancer. Next, a nomogram was constructed by integrating the prognostic signature and clinical characteristics, including TNM stage, age, gender, and the risk score. The AUC of time-dependent ROC curves, DCA plot, and concordance index plot consistently showed that the nomogram had the best prediction efficiency compared to any single factor for the 1-year, 3-year, and 5-year OS of colorectal cancer patients, which suggested that combining the nomogram with the risk score and clinical factors could efficiently predict the OS compared to a single factor.

The GO and KEGG pathway analyses were used to analyze the function of DEGs in the low- and high-risk groups. The GO pathway analysis showed that the DEGs were mainly enriched in extracellular matrix organization and extracellular structure organization, similar to the Notch pathway, via extracellular cell-cell communication. On the other hand, KEGG pathway enrichment analysis revealed that the DEGs were enriched in HPV, focal adhesion, proteoglycans in cancer, and extracellular matrix receptor interaction, which may not fit very well with the Notch pathway perhaps due to the low number of genes.

As described above, overexpression of *HEY1* is a poor prognostic factor in all CRC patients. Therefore, we knocked out *HEYL* to explore its function in CRC. Results of the CCK8 and colony formation assays showed that knocking out *HELY* significantly attenuated cell proliferation in HCT116 and RKO cell lines, suggesting that *HEYL* may be a negative factor in prognosis.

## Conclusions

In conclusion, this study established a prognostic 2-gene marker based on TCGA and GEO CORD&READ cohorts. Results showed that this gene signature is an independent prognosis predictor of colorectal cancer. A nomogram constructed by combining this 2-gene signature and clinical information could accurately predict the 1-year, 3-year, and 5-year OS of individual colorectal cancer patients, and this result was validated using in vitro experiments. Collectively, the findings of this study showed that the 2-NPRG markers can predict the prognosis of CRC patients, thereby enabling individualized treatment.

## Supplementary Information

Supplementary Material 1.

Supplementary Material 2.

## Data Availability

No datasets were generated or analysed during the current study.

## Abbreviations

AUC: Area under the curve
CCK-8: Cell Counting Kit-8
COPD: Chronic obstructive pulmonary disease
CRC: Colorectal cancer
CSCs: Cancer stem cells
DCA: Decision curve analysis
DEGs: Differentially expressed genes
EGFR: Epidermal growth factor receptor
EMT: Epithelial-mesenchymal transformation
GEO: Gene Expression Omnibus
GO: Gene ontology
HR: Hazard ratio
KEGG: Kyoto Encyclopedia of Genes and Genomes
LASSO: Least absolute shrinkage and selection operator
NPRG: Notch pathway related genes
OS: Overall survival
PCA: Principal component analyses
RNA-seq: RNA-sequencing
ROC: Receiver operating characteristic
sgRNA: Single-guide RNA
TCGA: The Cancer Genome Atlas
TGF-beta: Transforming growth factor-beta
TNM: Tumor-lymph node metastasis
t-SNE: t-distributed stochastic neighbor embedding
