# Supplementary figures and images for: A Notch signal pathway related gene signature predicts overall survival in colorectal cancer
Source: BMC Gastroenterol. 2025 Oct 8;25:704. doi: 10.1186/s12876-025-04292-1 (PMC12506287; doi:10.1186/s12876-025-04292-1)

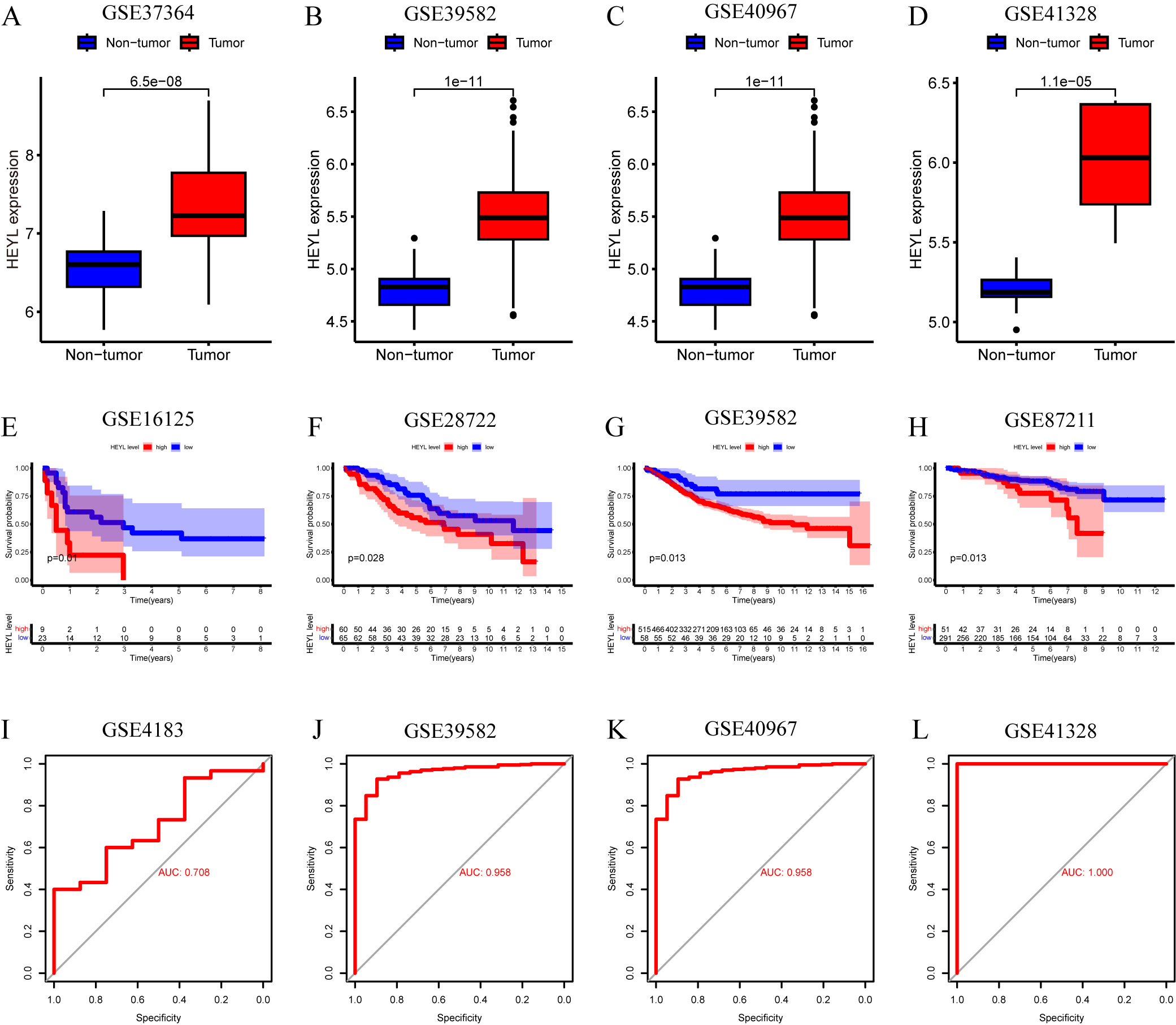

Supplement: Supplementary file 2 — Supplementary Material 2. [file 12876_2025_4292_MOESM2_ESM.tif]
